# Development and validation of a deep learning pipeline to measure pericardial effusion in echocardiography

**DOI:** 10.3389/fcvm.2023.1195235

**Published:** 2023-08-04

**Authors:** Chi-Yung Cheng, Cheng-Ching Wu, Huang-Chung Chen, Chun-Hui Hung, Tien-Yu Chen, Chun-Hung Richard Lin, I-Min Chiu

**Affiliations:** ^1^ Department of Computer Science and Engineering, National Sun Yat-sen University, Kaohsiung, Taiwan; ^2^ Department of Emergency Medicine, Kaohsiung Chang Gung Memorial Hospital, Kaohsiung, Taiwan; ^3^ School of Medicine, College of Medicine, I-Shou University, Kaohsiung, Taiwan; ^4^ Division of Cardiology, Department of Internal Medicine, E-Da Hospital, I-Shou University, Kaohsiung, Taiwan; ^5^ Division of Cardiology, Department of Internal Medicine, E-Da Cancer Hospital, I-Shou University, Kaohsiung, Taiwan; ^6^ Division of Cardiology, Department of Internal Medicine, Kaohsiung Chang Gung Memorial Hospital, Kaohsiung, Taiwan; ^7^ Skysource Technologies Co., Ltd., Taipei, Taiwan

**Keywords:** echocardiography, deep learning—artificial intelligence, pericardial effusion (PE), width measurements, automated segmentation, moving window (MW)

## Abstract

**Objectives:**

The aim of this study was to develop a deep-learning pipeline for the measurement of pericardial effusion (PE) based on raw echocardiography clips, as current methods for PE measurement can be operator-dependent and present challenges in certain situations.

**Methods:**

The proposed pipeline consisted of three distinct steps: moving window view selection (MWVS), automated segmentation, and width calculation from a segmented mask. The MWVS model utilized the ResNet architecture to classify each frame of the extracted raw echocardiography files into selected view types. The automated segmentation step then generated a mask for the PE area from the extracted echocardiography clip, and a computer vision technique was used to calculate the largest width of the PE from the segmented mask. The pipeline was applied to a total of 995 echocardiographic examinations.

**Results:**

The proposed deep-learning pipeline exhibited high performance, as evidenced by intraclass correlation coefficient (ICC) values of 0.867 for internal validation and 0.801 for external validation. The pipeline demonstrated a high level of accuracy in detecting PE, with an area under the receiving operating characteristic curve (AUC) of 0.926 (95% CI: 0.902–0.951) for internal validation and 0.842 (95% CI: 0.794–0.889) for external validation.

**Conclusion:**

The machine-learning pipeline developed in this study can automatically calculate the width of PE from raw ultrasound clips. The novel concepts of moving window view selection for image quality control and computer vision techniques for maximal PE width calculation seem useful in the field of ultrasound. This pipeline could potentially provide a standardized and objective approach to the measurement of PE, reducing operator-dependency and improving accuracy.

## Introduction

Pericardial effusion (PE) is a condition characterized by the accumulation of fluid within the pericardial space, which is typically diagnosed using transthoracic echocardiography. The buildup of fluid increases pressure within the pericardial sac, potentially leading to cardiac tamponade and decreased cardiac output. PE is a serious condition that requires timely intervention, making early detection and accurate measurement of the width of the effusion critical (1).

Echocardiography is considered the gold standard for the detection of PE due to its accessibility, portability, and ability to provide a comprehensive assessment of both anatomy and function (2–5). However, the presence and severity of PE can be uncertain, with mild effusion sometimes indicating pericardial fat rather than true effusion (6). Additionally, image quality can be compromised by factors such as breast tissue or obscuration from bone or lung. These challenges highlight the need for an accurate and reliable method for measuring PE that is not dependent on operator experience.

Artificial intelligence (AI) has been applied in various clinical settings to aid in the diagnosis of conditions based on echocardiograms, with considerable effort devoted to areas such as left ventricular function assessment, regional wall motion abnormality, right ventricular function, valvular heart disease, cardiomyopathy, and intracardiac mass (7–10). In particular, a study conducted in 2020 employed a deep learning model to detect PE in echocardiography and achieved an accuracy of 0.87–0.9 (11). In clinical practice, information on PE width and severity is crucial for initiating appropriate interventions. To the best of our knowledge, no study has yet analyzed the grading of PE using machine learning. Therefore, our study aimed to develop a deep learning model using echocardiography for PE detection and PE width measurement. Additionally, to facilitate the deployment of the deep learning model, we proposed an end-to-end guideline that can output detection results from raw ultrasound files.

## Method

The data collection and protocols utilized in this study were authorized by the Institutional Review Board of E-Da Hospital (EDH; no: EMRP24110N) and the Institutional Review Board of Kaohsiung Chang Gung Memorial Hospital (CGMH; no: 20211889B0 and 202101662B0).

### Data collection

In this study, images from routine echocardiography were generated at two medical centers, EDH and CGMH, in southern Taiwan. The deep learning model was trained and internally validated in EDH and externally validated in CGMH.

**Figure 1:**
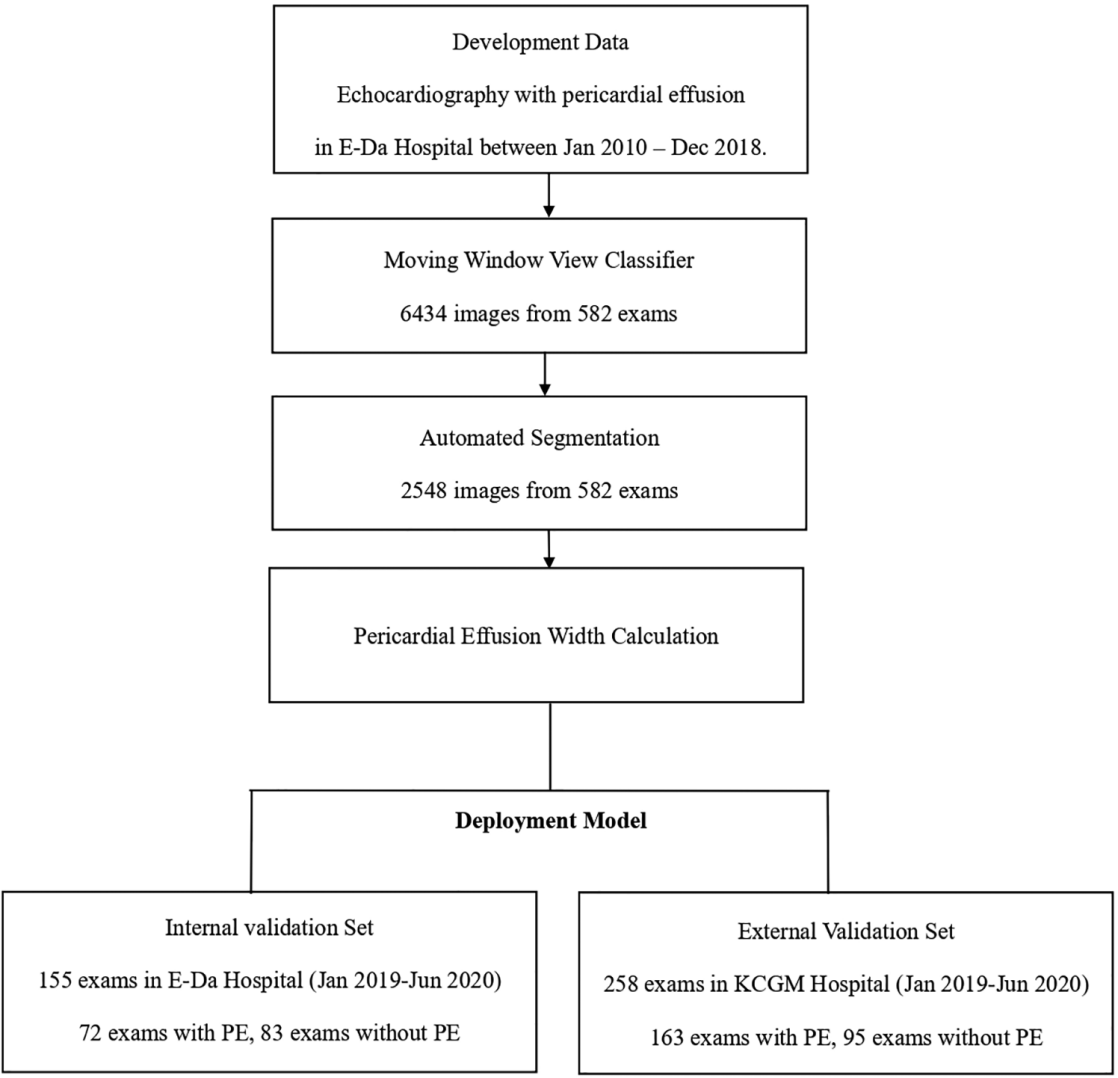
Flow chart illustrating the study design and data summary.

During the data collection process, we utilized the keyword “pericardial effusion” to search the Hybrid Picture Report System in EDH in order to gather a list of examination records. We obtained raw data from transthoracic echocardiography examinations with a diagnosis of pericardial effusion, which were performed at EDH between January 1, 2010, and June 30, 2020. These data were divided into training and validation datasets based on the respective examination dates. Examinations with dates prior to December 31, 2018, were used for the development of the model, and examinations with dates after January 1, 2019, were used for internal validation. To evaluate the generalizability of the model, we also retrieved echocardiography data from CGMH between January 1, 2019, and June 30, 2020, for external validation. The study flowchart and data summary are presented in Figure 1.

### Echocardiography

Images were gathered in a normal manner, with patients lying in the left lateral decubitus position. The ultrasound system (IE33, Philips Healthcare; S70, GE Healthcare; or SC2000, Siemens Healthineers) was used to perform echocardiographic examinations in EDH. Data from CGMH for external validation were acquired using EPIC7 (Philips Healthcare), Vivid E9 (GE Healthcare), or SC2000 (Siemens Healthineers). All examinations were saved in picture archiving and communication systems in the Digital Imaging and Communications in Medicine (DICOM) format.

After extracting the raw DICOM files, we processed the image from each patient to select the proper echocardiography views for developing a deep learning pipeline. The selected views were the parasternal long-axis (PLAX), parasternal short-axis (PSAX), apical four-chamber (A4C), and subcostal (SC) views. Two cardiologists manually measured the thickest point of pericardial effusion during each cardiac cycle as ground truth for width of PE. We employed ImageJ, an open-source software platform specifically designed for the scientific analysis and processing of images, to label and annotate the segmented masks corresponding to the echocardiography images. Upon completing the labeling process, the data was subsequently stored as CSV files on a secure, encrypted hard drive to ensure data integrity and confidentiality.

### Deep learning model development

**Figure 2:**
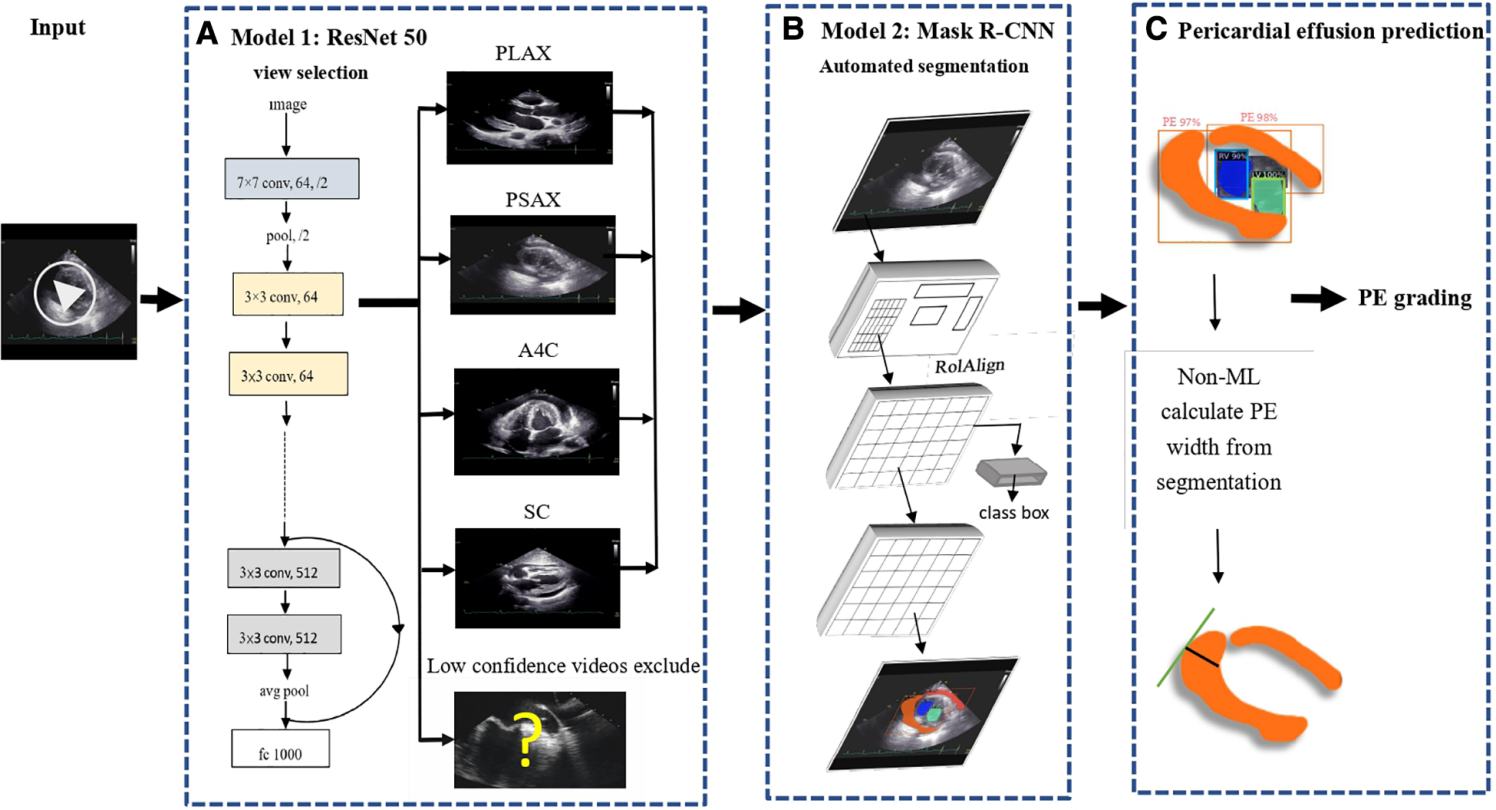
End-to-end pipeline for the automated measurement of pericardial effusion.

In this study, we developed an end-to-end pipeline for the automated measurement of PE based on the steps outlined below (Figure 2). The training subset of videos from EDH was used for the three main tasks of our pipeline:

### Step one: moving window view selection (MWVS)

We proposed a pipeline for managing echocardiography files directly from the workstation, similar to the work done by Zhang et al. and Huang et al., with some adjustments (Figure 2A) (10, 12). To distinguish the four primary views (PLAX, PSAX, A4C, and SC) from other views during each examination, we developed the first deep neural network model. This model was a ResNet-50-based two-dimensional model that aimed to classify each frame from the extracted DICOM files of echocardiography into the selected view types (13). To train this model, we randomly selected 6,434 images from the training dataset of EDH and labeled them according to the four primary views or other views, including low-quality views. We trained the model with a data split of 80% for the training set and 20% for the validation set. The model weight with the best detection performance in the validation set during the training process was preserved. The detection accuracy was assessed for each view class and weighted average result.

**Figure 3:**
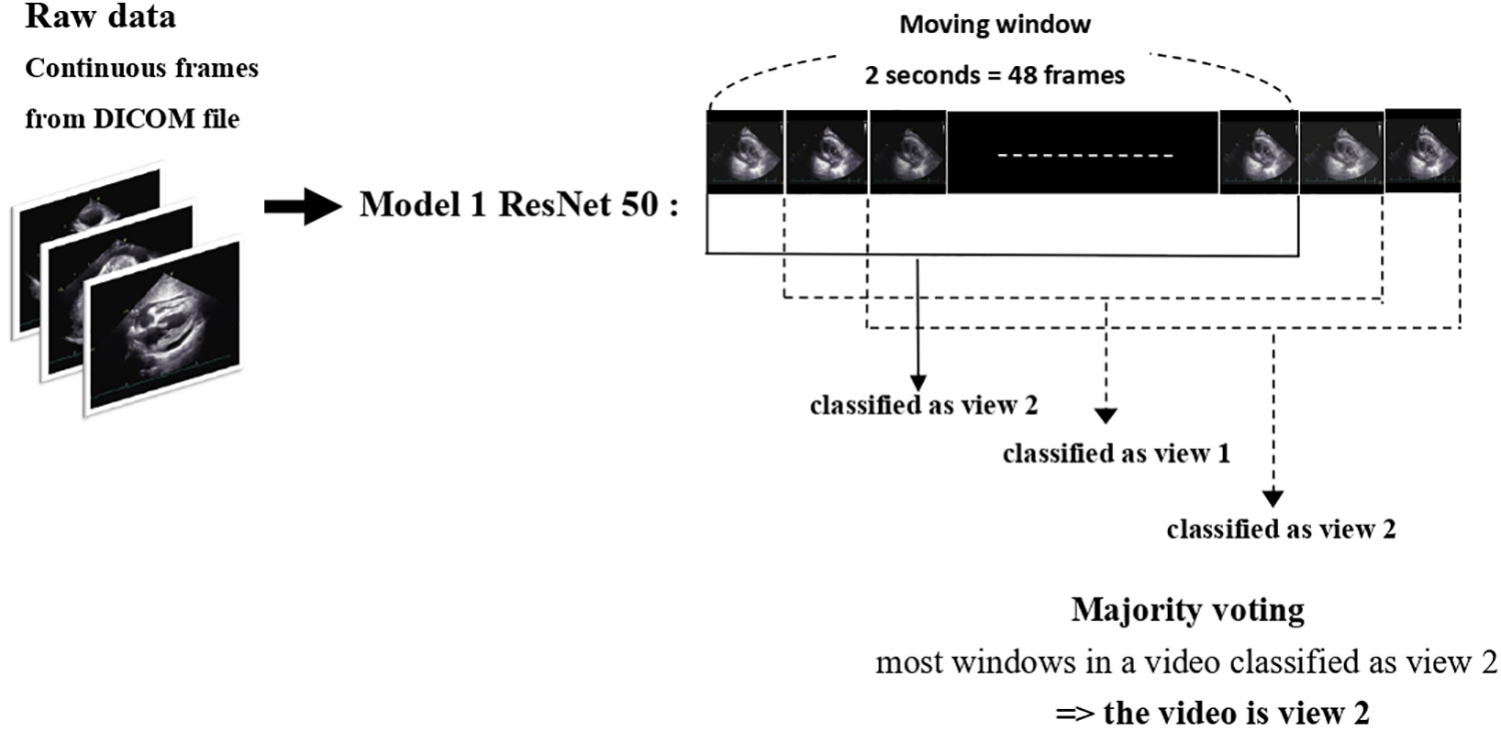
Moving window view selection concept.

In order to effectively manage the input video from patients during data collection, a 48-frame moving window was utilized to filter all videos. For each video, the best 48 frames with regard to specific view type and image quality were selected using a majority voting method (Figure 3). This process, known as MWVS concept, served not only as a view classifier but also as a quality control measure. Videos that did not contain at least 48 consecutive frames that met the image quality criteria of 50% or higher from one of the four primary views were excluded from further analysis. Additionally, the average view-classifying confidence levels for all images obtained from the selected 48-frame clip were used to evaluate the overall image quality and its correlation with performance. Videos with an average confidence level of less than 0.8 were also excluded from automated segmentation.

### Step two: automated segmentation

From the dataset, we randomly selected and annotated 2,548 frames in the EDH training dataset, ensuring an even distribution across the four primary views. We manually labeled the segmented area for pericardial effusion (PE) at three different phases in the cardiac cycle: end-systolic, end-diastolic, and the middle phase between the two aforementioned phases. The differentiation between epicardial adipose tissue and pericardial fluid was established. During this labeling phase, experienced clinicians manually segmented the area of PE based on its characteristic appearances in ultrasound images, while explicitly excluding epicardial adipose tissue. Additionally, we labeled the segmented areas for the four cardiac chambers to enhance the model's performance in separating these fluid-containing areas.

To train object instance segmentation based on the labeled ground truth, we utilized a Mask Region-Convolutional Neural Network (R-CNN) framework (Figure 2B). The model was trained with a data split of 80% for the training set and 20% for the validation set. Mask R-CNN is commonly used in medical applications for instance segmentation tasks as it can simultaneously perform pixel-level segmentation and classification of multiple target lesions (14). The implemented model generates bounding boxes and targeting masks for each instance of an object in an image. The input comprised consecutive ultrasound frames, and the output was a segmented mask indicating the corresponding four cardiac chambers and PE. The accuracy of the segmentation model was assessed using the Dice coefficient metric.

### Step three: measurement of pericardial effusion

After generating a segmented mask for pericardial effusion (PE), we proposed a computer vision technique, known as the maximal width calculator, to calculate the largest width of the PE in each ultrasound frame (Figure 2C). To accomplish this, we iterated through the vertical axis in each frame and hypothetically drew a horizontal line to determine if there was an intersection between the segmented mask and the horizontal line. If an intersection existed, we obtained a normal line from the edge of the mask over the intersection point. The length of the normal line that passed through the segmented mask was counted as the width of the PE at that intersection point. The largest width of the PE obtained through the iteration over the vertical axis was regarded as the width of the PE of the frame. This technique was applied to all 48 frames in the ultrasound video to provide the optimal PE width. Detailed explanation of this process was demonstrated in Supplementary Appendix 1.

### Statistical analysis

In this study, continuous variables are presented as either the mean and standard deviation if they are normally distributed or as the median and interquartile range if they are not. Dichotomous data are presented as numbers and percentages. The chi-squared test was used to analyze categorical variables, while continuous variables were analyzed using either the independent-sample *t*-test if they were normally distributed or the Mann-Whitney *U* test if they were not.

The performance of the proposed pipeline for PE width measurement was evaluated using metrics such as the mean absolute error, intraclass correlation coefficient, and R-square value when comparing the ground truth and detection. Additionally, the detection of the existence of PE and moderate PE was evaluated using sensitivity, specificity, and the area under the receiver operating characteristic curve. The deep learning models in the pipeline were developed using the TensorFlow Python package, image manipulation was performed using OpenCV 3.0 and scikit-image, and all analyses were conducted using SPSS for MAC version 26.

## Results

In this study, a total of 995 echocardiographic examinations were analyzed. Of these, 737 examinations were from the EDH dataset, with 582 being utilized for training and 155 for internal validation. Additionally, 258 examinations from the CGMH dataset were included for external validation. However, due to the limited number of SC views present in the CGMH dataset, this view was not included in the external validation analysis.

The demographic and clinical characteristics of the patients who underwent echocardiography are presented in Table 1. The mean age of patients in the training, internal validation, and external validation sets were 67.4 ± 15.4, 59.8 ± 19.2, and 66.4 ± 16.1 years, respectively. Additionally, 46.5% and 63.2% of patients in the internal and external validation groups, respectively, had PE. The average ejection fraction was 64.3 ± 7.1% in the internal validation group and 61 ± 13.9% in external validation group.

**Table 1:** Demographics, basic characteristics, and clinical findings of the patients.

Variables	Training set	Internal Validation set	External Validation set
Number of patients	582	155	258
Age(y)	67.4 ± 15.4	59.8 ± 19.2	66.4 ± 16.1
Gender	Male 49.5%	Male 53.2%	Male 58.1%
Height, cm	157.9 ± 9.2	162.1 ± 8.4	161.6 ± 8.4
Weight, kg	53.7 ± 35.6	65.9 ± 16.8	61.8 ± 13.3
BMI, kg/m^2^	23.3 ± 5.2	24.4 ± 1.5	23.6 ± 4.5
Mode EF %	61.1 ± 13.8	64.3 ± 7.1	61 ± 13.9
Mode EF < 50 (%)	12.8%	8.4%	19.3%
Patients with PE	582 (100%)	72 (46.5%)	163 (63.2)

The performance of the view classifier was evaluated with an average accuracy of 0.91 and 0.87 in predicting image classes in the training and validation sets, respectively. The independent accuracy in the validation set for each class was 0.90, 0.87, 0.93, 0.76, and 0.88 for PLAX, PSAX, A4C, SC, and others, respectively.

The MWVS was used to select the right views from all DICOM files in one examination and to improve video quality. The results of MWVS showed that 80%–100% of the four selected ultrasound views in EDH were successfully passed through for the segmentation model. In the external validation (CGMH dataset), 686 ultrasound videos from the four selected views were obtained. Our MWVS scanned through all DICOM files and 365 (53.2%) ultrasound videos were preserved for segmentation inference. The videos selected by our pipeline were further checked by a cardiologist, and none of them were misclassified as other cardiac views.

**Figure 4:**
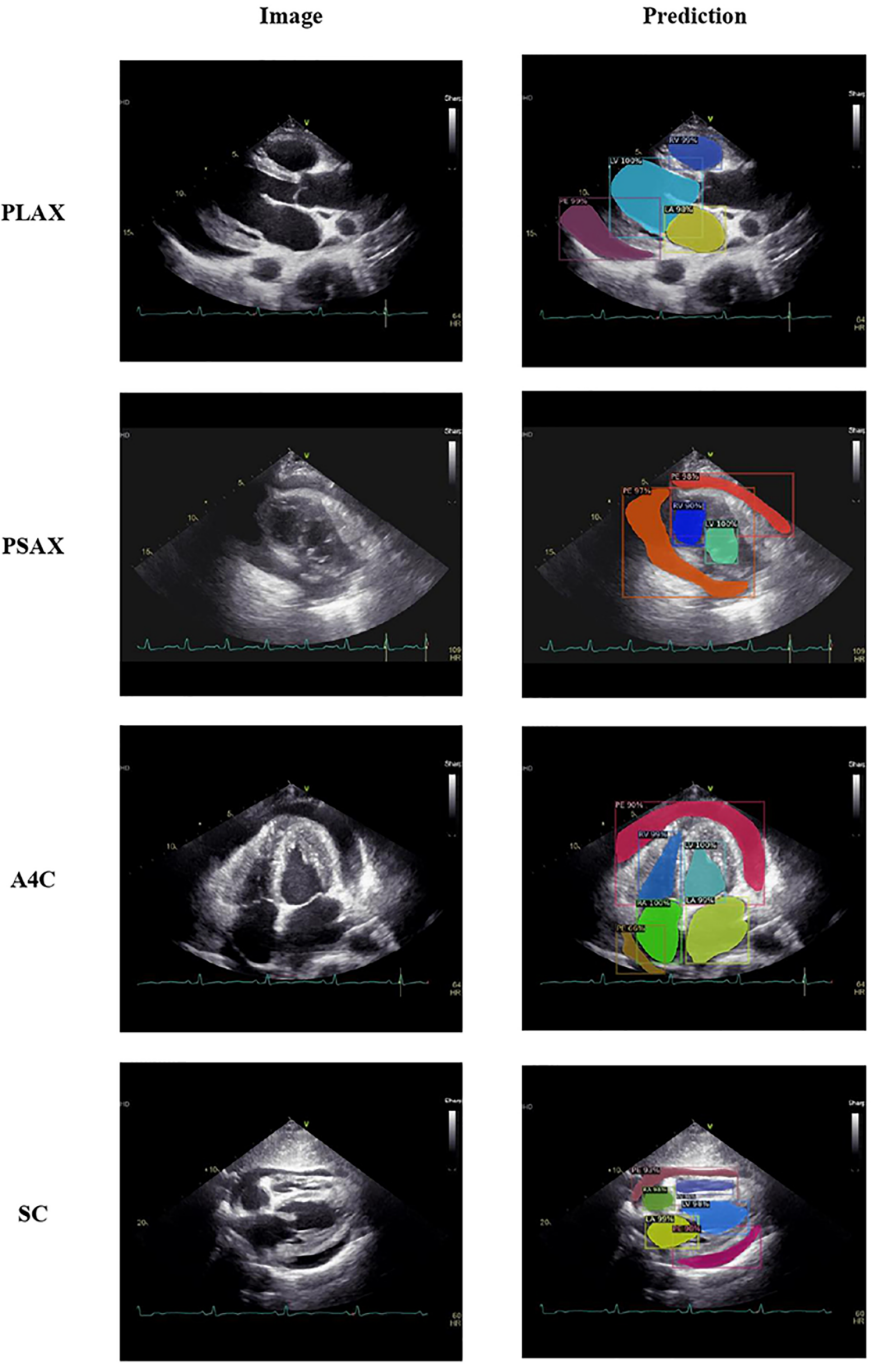
Image segmentation over cardiac chambers and pericardial effusion.

We employed a mask R-CNN-based model for image segmentation to effectively localize the cardiac chambers and PE area within four different views (Figure 4). The validation set, consisting of 510 images, showed an average Dice coefficient ranging from 0.67 to 0.82 among the four views, with the short-axis view (SC) achieving the lowest result. The best Dice coefficient was observed in the parasternal long-axis view (PLAX) at 0.72, while the SC view had the poorest result of 0.56 (Table 2). Using the segmented PE area, we calculated the maximal PE width from each frame.

**Table 2:** Dice coefficient of image segmentation.

	Dice coefficient					
	PE	RV	LV	RA	LA	Average
PLAX	0.72	0.86	0.85		0.84	0.82
PSAX	0.69	0.59	0.85			0.71
A4C	0.58	0.81	0.86	0.82	0.83	0.78
SC	0.56	0.66	0.71	0.70	0.72	0.67

**Figure 5:**
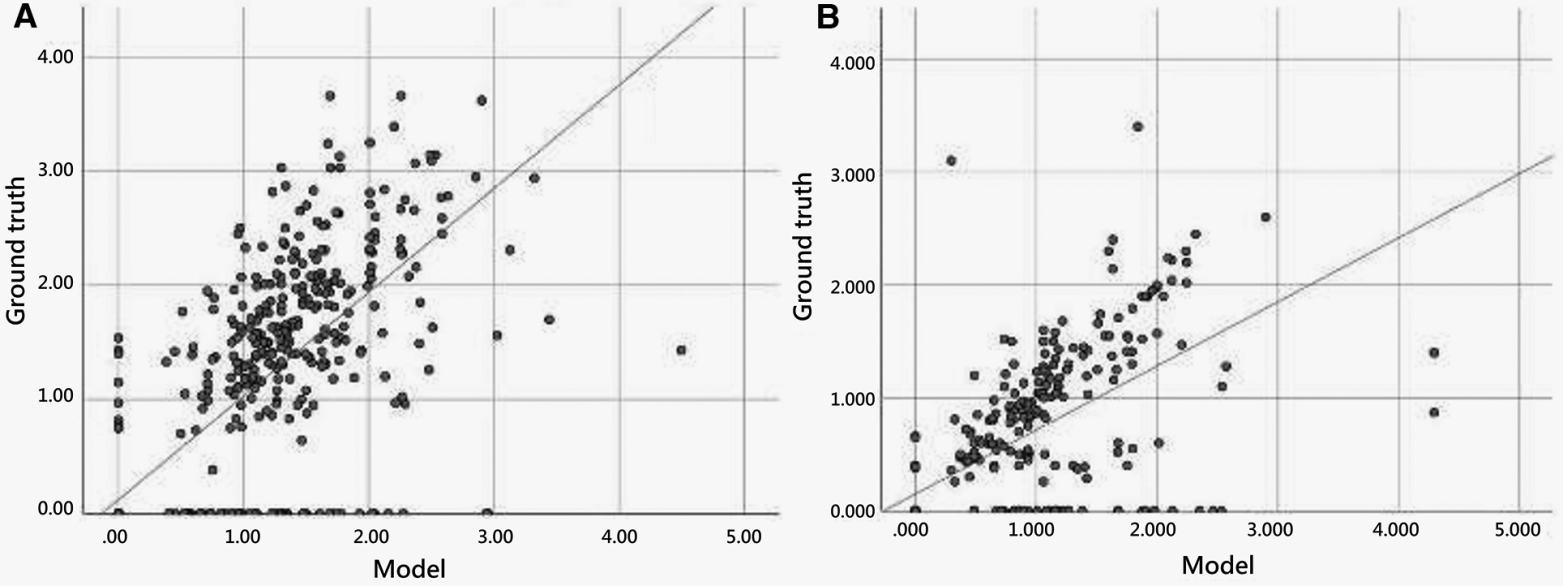
Scatter plot of pericardial effusion width measurement between model and manual annotation.

Figure 5 illustrates the scatter plot of the PE width measurement between the ground truth and model detection, which was determined by finding the largest normal line passing through the segmented mask in each frame. We compared the automated and manual measurements of PE width in both the internal (EDH) and external (CGMH) validation datasets, reporting the mean absolute error and correlation between the two. The mean absolute error was 0.33 cm and 0.35 cm in the internal and external datasets, respectively. Additionally, the interobserver variability was found to be highly correlated for the measurement of PE width between our model and human expert (ICC = 0.867, *p* < 0.001, EDH; ICC = 0.801, *p* < 0.001, CGMH). The R-square value was 0.594 for the EDH dataset and 0.488 for the CGMH validation dataset.

Our model accurately detected the existence of PE in the internal validation [AUC = 0.926 (0.902–0.951)] and external validation [AUC = 0.842 (0.794–0.889)]. With regard to recognizing moderate PE or worse, the AUC values improved to 0.941 (0.923–0.960) and 0.907 (0.876–0.943) in the internal and external validation groups, respectively.

We further performed a stratified analysis of the model detection in the different echocardiography views. In the internal validation, the model detection of PE width was highly correlated with the ground truth in the four different views, with ICC ranging from 0.802–0.910. The PLAX and A4C views appeared to have the best detection results with ICCs of 0.910 (0.876–0.935) and 0.907 (0.871–0.932), respectively. In the external validation, similar to internal validation, the model performed better in the PLAX and A4C views, with ICCs of 0.807 (0.726–0.864) and 0.897 (0.846–0.931), respectively. The other performances are listed in Table 3.

**Table 3:** Stratified analysis of the model detection in the different echocardiography views.

	Number before view selection	Number after moving window selection	Mean Absolute Error (cm)	ICC	R2
Internal validation					
PLAX	*n* = 155	*n* = 146	0.28	0.910 (0.876–0.935)	0.700
PSAX	*n* = 155	*n* = 155	0.46	0.802 (0.728–0.856)	0.469
A4C	*n* = 155	*n* = 155	0.32	0.907 (0.871–0.932)	0.754
SC	*n* = 155	*n* = 124	0.40	0.865 (0.808–0.905)	0.590
External validation					
PLAX	222	*n* = 127	0.32	0.807 (0.726–0.864)	0.457
PSAX	252	*n* = 138	0.44	0.714 (0.600–0.796)	0.337
A4C	212	*n* = 100	0.11	0.897 (0.846–0.931)	0.662

## Discussion

In recent years, computer vision and deep learning techniques have been utilized to aid in the interpretation of echocardiography, estimate cardiac function, and identify local cardiac structures. Deep learning algorithms have also been applied to facilitate the diagnosis of PE (15). Nayak et al. developed a CNN that detected PE in the apical four-chamber (A4C) and short-axis (SC) views with accuracies of 91% and 87%, respectively (11). In this study, we propose a deep learning pipeline that can process raw DICOM files from ultrasound and predict the PE width in a clinical setting. This pipeline combines two deep learning models and one technical calculation algorithm to accurately predict PE width. There have been few efforts to predict PE existence, with some studies being based on computed tomography scans (16, 17). To the best of our knowledge, this is the first video-based machine learning model to measure PE width using echocardiography. The correlation between the measurement of our model and human experts was high in both the internal and external validation datasets, with the best performance observed in the parasternal long-axis (PLAX) view. The inference speed of our model, using one graphics processing unit (NVIDIA RTX 3090), was approximately 30–40 s for one examination, which is usually faster than human assessment.

The present study introduces two novel concepts for echocardiography analysis: the MWVS and the maximal width calculator of the segmented mask. These methods are particularly important for real-world applications, particularly when working with relatively smaller datasets. Previous studies often rely on datasets manually selected by human experts during dataset cleaning, and use only “textbook-quality” images for training (18–21). In contrast, the present study proposes an analytical pipeline that can automatically analyze echocardiograms and be easily applied to personal devices or web applications, thus eliminating the need for expert sonographers or cardiologists. Madani et al. developed a CNN that simultaneously classified 15 standard echocardiogram views acquired under a range of real-world clinical variations, and the model demonstrated high accuracy for view classification (21). Similarly, this study used echocardiogram video clips obtained from the real world, taken for a variety of clinical purposes, including ejection fraction calculation, and detecting PE, valve disease, regional wall abnormality, cardiomyopathy, and pulmonary arterial hypertension. An initial screening model for view classification and quality control was developed. All raw images from the medical image database were input into the screening model, leaving a specific view of sufficient quality for diagnosis. Additionally, the “moving window” concept was used to retrieve only clips with 48 consecutive frames that fulfilled the image quality criteria. By avoiding limited or idealized training datasets, it is believed that this model is broadly applicable to clinical practice.

The method of MWVS is a novel concept that has not yet been proposed in the field of echocardiography assisted by machine learning. MWVS serves as an image quality filter and plays a crucial role in ensuring that the images are of sufficient quality for the next step in the pipeline. In the EDH dataset, echocardiography is performed by well-trained technicians who adhere to a protocol established by the echocardiologist consensus committee. As such, the original images from EDH were of relatively homogeneous quality, and MWVS filtered out fewer patients. Conversely, at CGMH, echocardiography is performed by individual echocardiologists who may have varying techniques. As a result, the original images from CGMH were less homogeneous, and MWVS filtered out more patients. This finding highlights the significance of MWVS in maintaining image quality and highlights the importance of image homogeneity in the applicability of machine learning models.

After segmenting the PE area, we developed a novel computer vision-based technique to calculate the largest PE width in ultrasound video. The current categorization of PE size relies on linear measurements of the largest width of the effusion at end-diastole, and is graded as small (<1 cm), moderate (1–2 cm), or large (>2 cm) (22). This semiquantitative classification method is prone to errors due to asymmetric, loculated effusions and shifts in fluid location during the cardiac cycle (23). Therefore, an automated calculation system could help identify the largest width of the PE in every ultrasound frame without any errors. In comparison to AI-based models, the computer vision technique is more similar to the method used by human experts. AI-based models not only consume more computing resources, but also require a large number of datasets for training and validation. To the best of our knowledge, our study is the first to not only detect but also classify the grade of PE.

Our model's ability to automate the process of classifying PE severity, traditionally categorized as mild, moderate, or severe, signifies a meaningful advancement in this field. Although this may appear straightforward for experienced clinicians, automated segmentation and measurement can be invaluable, especially in contexts where echocardiography expertise may be limited or entirely absent. Importantly, even though our model does not currently provide prediction on hemodynamic instability such as cardiac tamponade, its ability to differentiate moderate to severe PE from none or mild is crucial. This feature allows for early risk stratification in patients, prompting clinicians to initiate appropriate assessments and interventions as early as possible. Moreover, our work lays the groundwork for the future development of models designed to predict complex clinical scenarios, such as early hemodynamic instability. The integration of our model's segmentation and classification capabilities with other clinically relevant parameters may, in time, lead to major advancements in the prediction and management of such conditions.

This study had certain limitations that should be considered when interpreting the results. Firstly, the study was conducted retrospectively and the model was trained using data from only one hospital. This resulted in a limited sample size and a lack of ethnic diversity, which may impact the generalizability of the findings to other populations. To address this, future studies should utilize a multicenter design with greater heterogeneity in the dataset. However, it is important to note that the images used in this study were obtained using different ultrasonography machines and were interpreted by multiple echocardiographers, and the model achieved similar results during external validation. Secondly, while the proposed pipeline did grade the amount of PE, there was no information on whether there were signs of cardiac tamponade, as PE volume does not necessarily correlate with clinical symptoms (24). Additionally, due to the small sample size, we were unable to conduct subgroup analysis to distinguish the algorithm's performance on transudative vs. exudative effusions. To increase the clinical applicability of the findings, larger studies and validation cohorts are needed to reproduce the results of this study. Further research should also evaluate the collapsibility of the cardiac chambers and the presence of tamponade signs.

## Conclusion

In this study, we developed a deep-learning pipeline that automatically calculates the width of the PE from raw ultrasound clips. The model demonstrated high accuracy in detecting PE and classifying the PE width in both internal and external validation. The use of a novel concept, known as MWVS, for image quality filtering and computer vision techniques for calculating the maximal PE width is a novel application in the field of echocardiography.

## Data Availability

The raw data supporting the conclusions of this article will be made available by the authors, without undue reservation.
