# Supplementary material for: Development and validation of a deep learning pipeline to measure pericardial effusion in echocardiography
Source: Front Cardiovasc Med. 2023 Aug 4;10:1195235. doi: 10.3389/fcvm.2023.1195235 (PMC10436508; doi:10.3389/fcvm.2023.1195235)
Supplement: Supplementary file 1 [file Datasheet1.docx]

Appendix 1. Detailed Procedure for Computing the Maximal Width Across the Pericardial Effusion Area: A Step-by-Step Code Walkthrough

1. **get_pe_masks**: This function predicts the masks of pericardial effusion (PE) in the input image using a trained predictor. It returns all masks corresponding to the PE class with a confidence score greater than a certain threshold.

2. **get_tangent_and_normal**: Given two points (p1 and p2) on the PE mask, this function calculates the tangent and normal lines to the line passing through these two points. The tangent is calculated using the slope formula, and the normal is calculated as the line perpendicular to the tangent and passing through the midpoint of p1 and p2.

3. **get_y**: This function traverses the image horizontally from either left or right depending on the direction specified, and returns the y-coordinate of the first pixel that belongs to the PE mask. If no such pixel is found, it returns None.

4. **draw_pe_line**: This function draws the tangent and normal lines on the image. The tangent line is drawn in green and the normal line (indicating the PE thickness) is drawn in red. The length and direction of the normal line are specified in the function arguments.

5. **get_pe_width**: For each PE mask, this function calculates the maximum PE thickness at different points on the mask. For each point on the mask, it calculates the tangent line, then calculates the normal line to the tangent, and determines the PE thickness as the maximum length along the normal line that remains within the mask. It then returns the parameters of the normal line that results in the maximum PE thickness.

6. **pe_width_test**: This function reads an echocardiography image, predicts the PE masks, and calculates the maximum PE thickness for each mask. It also optionally displays each mask with the tangent and normal lines drawn on it.
